# Effects of Thigh and Leg Rotation on Sagittal Knee Angle During Static Assessment

**DOI:** 10.3390/jfmk10030235

**Published:** 2025-06-20

**Authors:** Bruna Nichele da Rosa, Edgar Santiago Wagner Neto, Matias Noll, Jefferson Fagundes Loss, Cláudia Tarragô Candotti

**Affiliations:** 1 Escola de Educação Física, Fisioterapia e Dança, Universidade Federal do Rio Grande do Sul, Porto Alegre 90690-200, Brazil; edgar.wagner@ufrgs.br (E.S.W.N.); jefferson.loss@ufrgs.br (J.F.L.); claudia.candotti@ufrgs.br (C.T.C.); 2 Departamento de Nutrição, Universidade Federal de Goiás, Goiânia 74690-900, Brazil; 3 Instituto Federal Goiano, Campus Ceres, Ceres 76300-000, Brazil

**Keywords:** knee, rotation, posture

## Abstract

**Background:** The femur and tibia can suffer changes in rotation, mainly in the orthostatic position, causing errors in measurements when two-dimensional instruments are utilized. Objectives: To test the effects of thigh and leg rotation on sagittal knee angle measurement. **Methods:** A physical model simulating the right lower limb was constructed using two wooden rafters and a plastic protractor between the rafters. The thigh rotation and leg rotation were measured, ranging from 50° of internal rotation to 50° of external rotation. The sagittal knee angle was measured using the three-dimensional kinematics via three protocols (femoral condyle angle, head of fibula angle, and four points angle) with points marked on the model corresponding to the greater trochanter of the femur, lateral condyle of the femur, head of the fibula, and lateral malleolus. **Results:** During the internal rotation of the thigh and leg, the sagittal knee angle increased (varying from 3.1° to 6.3° for thing, and 0.9° to 3.6° for leg), whereas it decreased during the external rotation of the thigh and leg (varying from −5.2° to −7.8° for thing, and 0.1° to −5.5° for leg). **Conclusions:** Thigh rotation and leg rotation affect sagittal knee measurement and can lead to erroneous assessments if not considered.

## 1. Introduction

The postural assessment of lower limbs is important for the diagnosis of misalignments and imbalances related to orthopedic and rheumatological conditions [1,2,3]. For example, the knee alignment on the frontal plane affects the load distribution on the joint, because valgus and varus misalignments increase the loads on the medial and lateral compartments, respectively [4,5,6]. The increase in load is a risk factor for progressive degeneration and knee osteoarthritis and is correlated to the increase in tension on the anterior cruciate ligaments, collateral ligaments, and joint capsule. In addition, it is associated with patellofemoral pain syndrome and iliotibial band syndrome [4,7,8,9,10].

In the case of sagittal plane alignment, we expected the full extension of the knee, which allows the weight to be supported without constantly expending muscular energy, and this alignment plays an important role in the maintenance of orthostatic balance [11,12]. When a misalignment in flexion (genum flexum) occurs, there is an increase in quadriceps activity to improve knee stability during weight bearing, which can lead to knee pain [11]. Additionally, this misalignment has functional effects on gait, e.g., reducing the gait speed and step length [13].

Static postural assessment of the knee can be performed by using the gold standard (X-ray exam) or alternative methods such as goniometry and photogrammetry. However, these alternative methods exhibit inconsistency because they are based on anatomical reference points marked on the skin’s surface [14,15,16,17,18]. The most common errors are related to the location, palpation, and marking of anatomical points [19]. Moreover, when analyzed using 2D methods, these reference points provide a measurement corresponding to the projection of the points on the sagittal plane, and they are not necessarily representative of the real angle between the structures, because the instruments consider only one plane in the assessment.

In photogrammetry, to measure the sagittal knee alignment, most protocols use three anatomical reference points: the greater trochanter of the femur (GTF), the lateral condyle of the femur (LCF), and the lateral malleolus (LM) [14,20,21,22]. However, the thigh and the leg can suffer internal or external rotation [23], which can move the anatomical reference points from a sagittal point of view, leading to erroneous sagittal knee angle measurement. The displacements of the anatomical points affect the sagittal knee measurement. Therefore, in the sagittal view, when a two-dimensional instrument is used for assessment, as in the case of photogrammetry, errors caused by the inability to visualize the 3D changes in body segments can occur. Consequently, the knee alignment on the sagittal plane can be erroneously classified when a change on the transversal plane occurs, which can be a source of inconsistency in this assessment method. From this perspective, the objective of the present study was to test the effects of thigh and leg rotation on sagittal knee angle measurement using a physical model simulating the lower limb, with different anatomical reference points.

## 2. Methods

### 2.1. Physical Model

**Figure 1:**
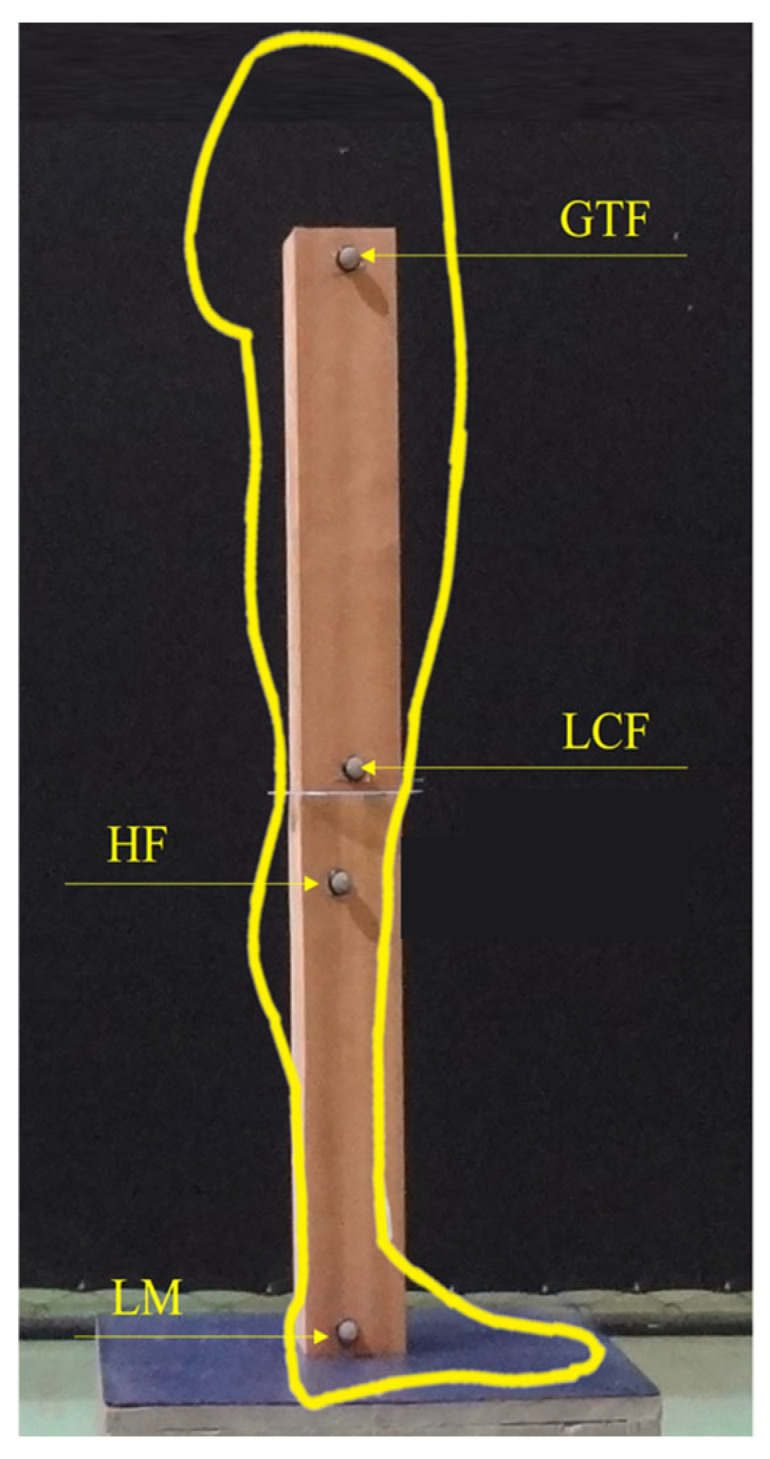
Developed a physical model with rafters simulating the right thigh and leg, and the reflexive plastic markers corresponding to the anatomical reference points. GTF, greater trochanter of the femur; LCF, lateral condyle of the femur; HF, head of the fibula; LM, lateral maleollus.

A physical model simulating the right lower limb was built with two wooden rafters, each having a length of 40 cm and a width of 8 × 8 cm^2^. The rafters were positioned one above another. The upper rafter simulated the thigh, and the lower rafter simulated the leg of a person undergoing static postural assessment of the knee in the sagittal plane (Figure 1).

Reflexive plastic markers were positioned at locations corresponding to the following anatomical points: the GTF, LCF, head of the fibula (HF), and LM (Figure 1). The markers were positioned according to the lower limbs anatomical parameters of an adult [24], based on the measurements made by Sharma et al. [25], with both the thigh and leg 40 cm long.

**Figure 2:**
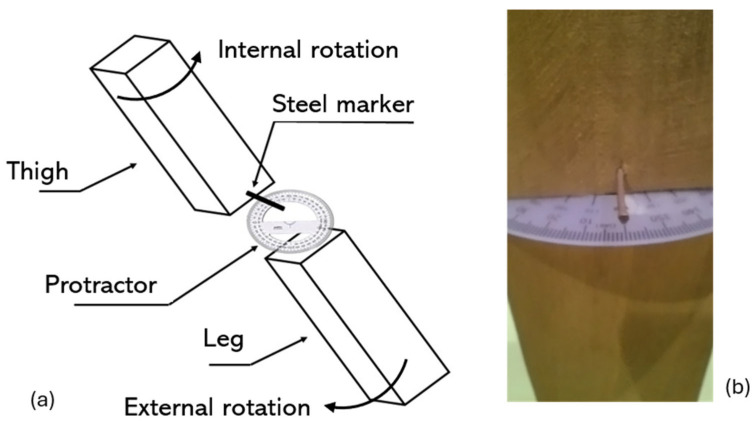
Rafters simulating the right thigh and leg and the protractor between the two rafters to measure the rotation simulating the positions of the thigh and leg: (**a**) schematic of physical model; (**b**) frontal image.

To measure the thigh rotation and leg rotation during each assessment, a plastic protractor was positioned between the two rafters, and a steel marker was fixed in the rafter that simulated the thigh (Figure 2), to mark the angle of rotation to be tested. Internal and external rotation of the thigh and the leg were simulated with increments of 10°. Therefore, we simulated the postural assessment of an adult presenting torsional changes in the thigh and leg, which varied from 50° of internal rotation to 50° of external rotation, in accordance with the findings of Tamari et al. [23], who assessed healthy adults via magnetic resonance imaging. In total, 21 static assessments of the sagittal knee angle were performed using the model: 10 simulating an adult with thigh rotation (five of thigh internal rotation of 10°, 20°, 30°, 40°, and 50°, and five of thigh external rotation of 10°, 20°, 30°, 40°, and 50°), 10 simulating an adult with leg rotation (five of leg internal rotation of 10°, 20°, 30°, 40°, and 50°, and five of leg external rotation of 10°, 20°, 30°, 40°, and 50°), and the lower limbs neutral position (without thigh or leg rotation). When a segment (thigh or leg) simulating internal or external rotation was positioned, the other segment (thigh or leg) remained in a neutral position (0° of rotation). In all the simulations, the alignment between the upper and lower rafters was 180°; that is, the simulations focused only on the transversal plane between the thigh and leg.

### 2.2. Knee Flexion Angles Protocols

**Figure 3:**
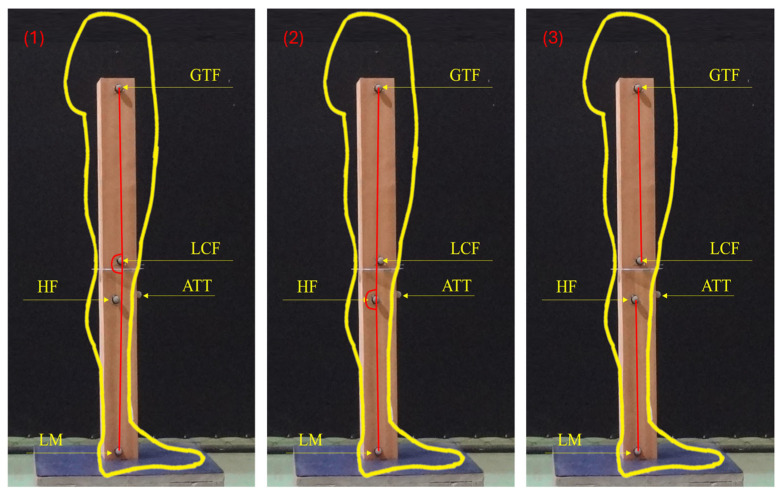
Sagittal knee angles measured: (**1**) Femoral condyle angle (CA) formed by the GTF, LCF, and LM; (**2**) Head of fibula angle (HFA) formed by the GTF, HF, and LM; and (**3**) Four points angle (4PA) formed by the prolongation of the line formed by the GTF and LCF and the line formed by the HF and LM.

Static assessment of the knee was performed via 3D kinematics using a BTS Smart-DX system (BTS Bioengineering, Milan, Italy) with 10 infrared cameras, with a sampling rate of 100 Hz. The cameras were positioned three meters high in a half-circle around the physical model to allow visualization of all points by all cameras. The system was calibrated according to the manufacturer’s specifications prior to data collection, achieving an error margin of less than 0.3 mm for all points within the calibration volume. Three-dimensional kinematics was used because of the ability to employ different analysis protocols, considering different anatomical reference points for identifying possible influences of thigh and leg rotation in each protocol. On the basis of 3D kinematics, the following three protocols were used to measure the sagittal knee angle: (1) the posterior angle of the lower limb formed by the GTF, LCF, and LM, which is called the “femoral condyle angle” (CA); (2) the posterior angle of the lower limb formed by the GTF, HF, and LM, which is called the “head of fibula angle” (HFA); and (3) the posterior angle of the lower limb formed by the prolongation of two lines—one formed by the GTF and LCF and another formed by the HF and LM—which is called the “four points angle” (4PA) (Figure 3). Considering the orthostatic position, the most used in the protocols of postural assessment using photogrammetry [22], only the neutral knee extension position was tested.

### 2.3. Data Analysis

Since all measurements were static, the kinematic data were used as raw data without any filtering. The kinematic data of each position was assessed for three seconds, and the central second (100 points) was used to represent the situation. The simple arithmetic mean of these 100 central points was used to obtain each angle. The sagittal knee angles resulting from the analysis were tabulated. A scatter plot was generated with all the data using Microsoft Excel (version 2019, Microsoft Corporation, Washington, DC, USA). The internal rotations of the thigh and leg were represented by negative values in the plot, and the external rotations of the thigh and leg were represented by positive values. Additionally, the mean difference relative to the neutral position (without any rotation of the thigh or leg) was calculated for five external rotations of the thigh and leg (10°, 20°, 30°, 40°, and 50°) and five internal rotations of the thigh and leg (10°, 20°, 30°, 40°, and 50°).

## 3. Results

**Figure 4:**
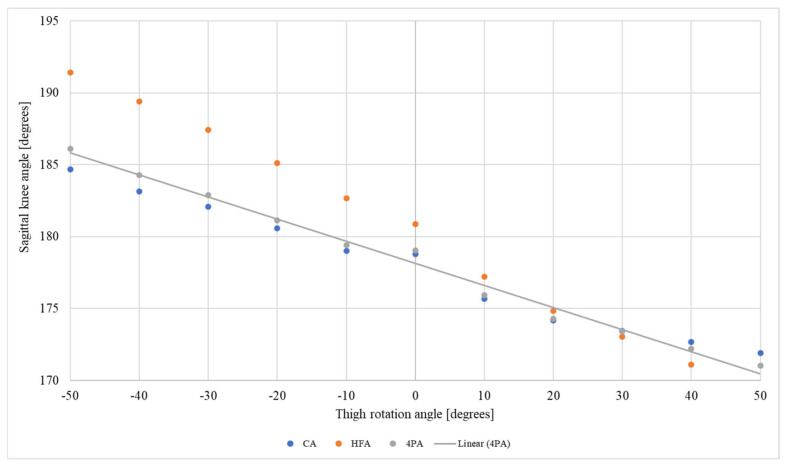
Sagittal knee angle obtained from each analysis protocol (CA, femoral condyle angle; HFA, head of fibula angle; 4PA, four points angle) for each of the thigh rotations tested (negative values correspond to internal rotation, and positive values correspond to external rotation).

Figure 4 presents the sagittal knee angle measurements based on each protocol used, which simulate the thigh positions (neutral, internal, and external rotations). Regardless of the protocol used and therefore of the anatomical points used in the calculation, the sagittal knee angle exhibited the same behavior. There was an increase in the knee angle during the increments of thigh internal rotation, that is, a false interpretation of knee hyperextension. During the increments of thigh external rotation, there was a reduction in the sagittal knee angle (Figure 3). Depending on the protocol used and the type of rotation, differences of >10° in the sagittal knee angle were measured.

**Figure 5:**
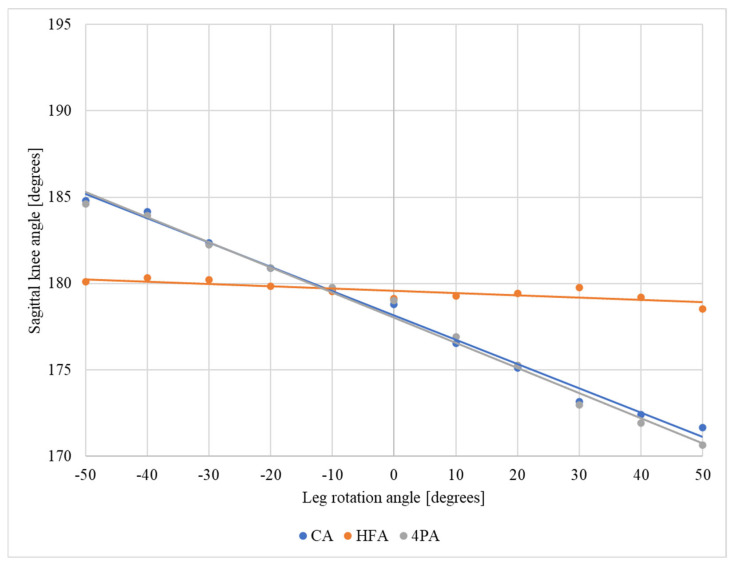
Sagittal knee angle obtained from each analysis protocol (CA, femoral condyle angle; HFA, head of fibula angle; 4PA, four points angle) for each of the leg rotations tested (negative values correspond to internal rotation, and positive values correspond to external rotation).

Regarding the leg position simulation (neutral position, internal rotation, and external rotation) (Figure 5), the behavior of the sagittal knee angle during the leg rotation was identical among all the protocols except the one using the points on the GTF, HF, and LM (“head of fibula angle”). The values increased during the internal rotation increment, that is, with a false interpretation of knee hyperextension. Additionally, the values decreased during the external rotation, with a false interpretation of knee flexion. The “head of fibula angle” protocol exhibited a stable sagittal knee angle of approximately 180°, independent of the leg position. The results indicated that depending on the protocol used and the type of rotation, distortion of approximately ±9° occurred.

Table 1 presents the mean values of the change in sagittal knee angle measurement, considering all the tests with external and internal rotation of the thigh and leg.

## 4. Discussion

As researchers in the field of photogrammetry with over 20 years of experience, we frequently observe erroneous interpretations related to the knee sagittal angle using photogrammetry during rotations in the transversal plane in thigh and leg segments. The test involving the simulation of the right lower limb using the physical model with two rafters—one representing the thigh and the other representing the leg—and with a plastic protractor representing the knee revealed that changes in the transversal plane (rotations) affect the sagittal knee alignment measurement. Except HFA protocol for leg rotation, we observed that in general, the knee angle behavior was similar regardless of whether the thigh or leg was internally or externally rotated.

As the thigh and leg rotate internally (Figure 4 and Figure 5), the sagittal knee angle increases, leading to a false interpretation of knee hyperextension. This false increase in the angle of the knee can lead to the alignment of the knee being wrongly classified as genu recurvatum, as the angle of the knee in the sagittal plane can exceed 185° [26], according to the leg or thigh rotation. Similarly, the alignment of the sagittal knee angle can be mistakenly classified as genu flexum [26] when there is external rotation of the thigh or leg. This is because as the thigh and leg externally rotate, there is a reduction in the sagittal knee angle, which can reach values of <170°, depending on the degree of rotation.

However, the alterations observed in the sagittal knee angle during the assessments do not correspond to true changes in the sagittal plane; for instance, as the rafters were not moved to simulate knee flexion or extension. The observed variations are attributable to measurement inaccuracies resulting from the distinct positioning of anatomical markers due to torsional variations in the transversal plane. Torsional changes in the thigh region, as depicted in Figure 4, result in the anterior or posterior displacement of the anatomical marker on LCF, potentially influencing the sagittal knee measurements. A similar effect is observed in the leg region due to torsional changes, as illustrated in Figure 5, where the marker on HF and LM shifts anteriorly or posteriorly, thereby affecting the sagittal plane measurements.

The changes in the sagittal knee angle measurements occurred regardless of the protocol used and, therefore, the anatomical reference points considered for the calculation. Existing protocols in photogrammetry—the main method for quantitatively assessing the static alignment of body segments [27,28]—employ the GTF, LCF, HF, and LM as anatomical reference points because they are bony prominences that are easy to palpate [14,15,20,21]. According to the results of our study, regardless of the protocol used to measure the knee angle in the sagittal plane, there is a measurement error associated with possible rotations of the thigh, leg, or both.

The “head of fibula angle” protocol was the only one that proved to be stable; however, it was stable only when leg rotations occurred (Figure 5), as the maximum variation in the knee angle in the sagittal plane was 1.2° at 40° of internal leg rotation. For the other protocols in the case of leg rotations, as well as for all the protocols in the case of thigh rotations, there were considerable variations in the knee angle in the sagittal plane. The largest average variations in the knee angle during thigh rotations were 10.5° for internal rotation and 11.4° for external rotation. Compared with thigh rotations, the variations were smaller in magnitude for leg rotations; the largest variations were 6° for internal rotation and 8.4° for external rotation.

Importantly, in our study, knee angles were measured in the sagittal plane, simulating rotation in only one of the segments that constitute the knee joint: either in the thigh while the leg remained neutral or in the leg while the thigh remained neutral. However, when an individual is assessed, these rotations can be present together [23,29], which can lead to additional measurement errors. In a similar way, these transversal changes can occur in parallel with changes in other planes, such as knee varus or knee valgus. Consequently, further investigation is warranted to elucidate these aspects. Moreover, existing methods for quantifying knee alignment in the sagittal plane should consider the measurement errors that can occur with changes in the transverse plane, thereby preventing erroneous assessments.

Despite the study’s limitations, it is important to emphasize that the findings demonstrate the impact of thigh or leg rotations on knee angle measurement in the sagittal plane using a physical model. However, the differences in geometry between the physical model and the human body prevent us from extrapolating the differences in sagittal knee angle to real situations. Therefore, our findings cannot be used for knee angle corrections using photogrammetry. More representative models of the human body must be used for this purpose. The results presented in this study are a first step in this direction, highlighting the potential for error when lower limb rotations are not considered during bi-dimensional sagittal knee angle measurement.

This contributes to the ongoing discourse on the significance of assessing all planes during knee evaluation. It is known that tibiofemoral joint stability encompasses both ligaments and muscles, providing dynamic stability [30]. In professional athletes, particularly, imbalances in muscular strength can generate torsional changes, contributing to misalignment in other planes and the development of injuries and disability [6,7].

## 5. Conclusions

From the measurements performed on the physical model using rafters simulating the right lower limb, it can be concluded that thigh and leg rotations could influence the measurement of the knee angle in the sagittal plane. In the physical model tested, the knee angle increases or decreases depending on the degree and type of rotation (internal or external) of the thigh and leg, owing to the displacements of the anatomical points used as references for the calculation. The results obtained cannot be used for possible corrections in measurements of the sagittal knee angle in humans. However, our findings may explain some of the inconsistencies found in photogrammetric knee assessment methods that use the anatomical landmarks tested (GTF, LCF, HF, and LM).

## Figures and Tables

**Table 1:** Mean difference relative to the neutral position for simulated rotation in the physical model.

Protocol	Mean Difference Relative to the Neutral Position—Thigh		Mean Difference Relative to the Neutral Position—Leg	
	Internal Rotation	External Rotation	Internal Rotation	External Rotation
Condyle angle (CA)	3.1°	–5.2°	3.6°	–5°
Head of fibula angle (HFA)	6.3°	–7.8°	0.9°	0.1°
Four points angle (4PA)	3.7°	–5.7°	3.3°	–5.5°

## Data Availability

The data presented in this study are available on request from the corresponding author. The data is not publicly available as we do not have a public repository. But if you are interested, please contact Bruna (bruna.nichele@gmail.com) and we will send you the information.
